# The Role of Entrepreneurship Policy in College Students’ Entrepreneurial Intention: The Intermediary Role of Entrepreneurial Practice and Entrepreneurial Spirit

**DOI:** 10.3389/fpsyg.2021.585698

**Published:** 2021-03-01

**Authors:** Yangjie Huang, Lanyijie An, Jing Wang, Yingying Chen, Shuzhang Wang, Peng Wang

**Affiliations:** ^1^Institute of Innovation and Entrepreneurship Education, Wenzhou Medical University, Wenzhou, China; ^2^Department of Humanities and Communication, Wenzhou Polytechnic, Wenzhou, China

**Keywords:** entrepreneurship policy, entrepreneurial spirit, entrepreneurial intention, entrepreneurial practice, entrepreneurship education, empirical study

## Abstract

Entrepreneurship is a sustainable development tool that supports the alleviation of poverty and unemployment. Focusing on the promotion of entrepreneurial intention (EI) under the background of entrepreneurship education (EE), this study used a structural equation model to examine the role of entrepreneurship policy (EPo), entrepreneurial practice (EPr), and entrepreneurial spirit (ES) on the EI of 384 college students from 22 universities in Guangdong Province. The test results show that there are significant positive correlations between EPo and EI; EPo and EPr; EPo and ES; and EPr and EI. They also support the hypothesis that EPr enhances the positive effect that EPo has on EI. This study puts forth measures to improve EI and makes contributions to future research on EE.

## Introduction

Since the dawn of the 21st century, the world has attached more importance to sustainable development. Business enterprises and entrepreneurs play an important role in sustainable development (Pizzi et al., 2020; Veleva, 2020) from the poverty and unemployment reduction perspective, whereas entrepreneurship, to some extent, can be interpreted as the process of establishing a new enterprise (Gartner, 1985). Entrepreneurship has become an important vehicle for sustainable development: helping to increase employment and promote economic growth (Urbano et al., 2020).

Education is also linked to sustainable development. In 2015, the United Nations placed education at the heart of its sustainable development strategy and many schools now incorporate sustainability education into their curriculum (Annan-Diab and Molinari, 2017). Individual entrepreneurial activities are the process from idea to practice, that is, from intention to behavior. There is a positive correlation between entrepreneurial intention (EI) and entrepreneurial behavior (Kong et al., 2020). When coupled with EI, entrepreneurship education (EE) can promote students’ entrepreneurial behavior (Ginanjar, 2016). Along with flexible and coherent public policies, EE is conducive to entrepreneurship (Bagiatis et al., 2019). EE and policy are therefore, key considerations. Researching the influence of entrepreneurship policy (EPo) EI can help us to understand whether EPo can improve the interest of students receiving EE, and how to better stimulate their EI, with the aim of reducing the employment pressure faced by college students.

Following the advent of emerging technologies, many industries have become more complex and are characterized by the requirement for increasingly diverse capabilities. In this global innovation environment, entrepreneurship is a challenge. For college students in particular, competition is increasingly fierce, and navigating the challenges of entrepreneurship has become more difficult. Unfortunately, the pedagogy of innovation and entrepreneurship in schools has yet to come of age. There is little literature that further elaborates the intermediary mechanism between EPo and EI. Thus, to better equip college students to start businesses and develop entrepreneurial talents, it is imperative for the academic community to develop the practice of EE more intensively. This study used the EI of university students in the Guangdong Province, China, as the basis for its investigation and research. It explores the role of EPo, ES, and EPr in EI and also considers the intermediary role of EPr (coupled with ES), on EPo and how this may influence EI.

## Literature Review and Hypotheses

### Entrepreneurship Education

Entrepreneurship education aims to impart entrepreneurship knowledge, skills, and experience to students through entrepreneurship courses, entrepreneurship competitions, and other training, to cultivate innovative thinking and entrepreneurial ability. The development of EE began in 1947 when Harvard Business School opened the first EE course, “Management of New Enterprises,” and has continued to develop gradually since then. EE in colleges guides students to exercise their comprehensive abilities such as the ability to identify opportunities, the ability to innovate, and psychological strength. For example, it is helpful for students wishing to succeed in entrepreneurship to engage in a series of EE experiences and cultivate their analytical ability and intuition (Raposo and Do Paco, 2011). In the current era of rapid scientific and technological development, EE can cultivate more entrepreneurial talents with innovative spirit, create more enterprises, adapt to the trends of the times, and promote economic development (Henry et al., 2003).

### Entrepreneurial Intention

This study focuses on EI in the context of EE. Entrepreneurship is a kind of planned behavior (Yi, 2018). According to the psychological theory of planned behavior (Ajzen, 1991), it can be predicted by the intention of this behavior. EI is the subjective attitude of potential entrepreneurs about whether they want to engage in entrepreneurial activities. It precedes entrepreneurship (Maâlej and Cabagnols, 2020) and is a necessary condition for entrepreneurship to occur. The higher the individual’s intention, the higher their entrepreneurial disposition (Kong et al., 2020). EI is influenced by the individual entrepreneur as well as the contingent environment that surrounds them. Personality traits like extroversion and emotional stability can affect EI indirectly through entrepreneurial self-efficacy (Mei et al., 2017). Attitude toward entrepreneurship (Law and Breznik, 2016), the need for achievement, and the inclination toward risk-taking (Popescu et al., 2016) all significantly influence EI. External factors like EE can cultivate more entrepreneurs (Asimakopoulos et al., 2019; Boldureanu et al., 2020) by identifying young people’s entrepreneurial interests early and developing them further (Escolar-Llamazares et al., 2019). A Malaysian empirical study illustrates that EE and EI are positively correlated (Abdullah et al., 2014) and a South African study finds that EE is closely related to the antecedents of EI (Michelle and Tendai, 2016). Paco et al. (2015) also suggest that other factors besides education will influence EI. Empirical research under different circumstances is of great significance to the aim of strengthening college students’ EI through EE.

### Entrepreneurship Policy

Entrepreneurship policy is comprised of actionable macro-, meso-, and micro-strategies implemented by the government to promote entrepreneurship. Academic circles only began to pay attention to EPo in the mid-1990s, and there is as yet no authoritative definition (Zhao and Li, 2011). Collins (2003) defined EPo as the policies and supporting measures adopted by the government to promote the establishment and growth of small enterprises. Globally, government policies are increasingly inclined to promote entrepreneurship (O’Connor, 2012). Hannon (2006) points out that the development of entrepreneurship is the center of many government policies because entrepreneurship can promote sustainable economic and social development. College students, as the most likely potential entrepreneurs, often give up entrepreneurship because of the lack of venture capital, financing, and policy support.

### Entrepreneurial Practice

Entrepreneurial practice (EPr) comprises a series of activities in which entrepreneurs engage to further their business ventures. Studies have shown that entrepreneurship is not an overnight process. Instead, there is a buffer period (Buttler and Sierminska, 2020) before achieving full entrepreneurship. During this time, necessary and practical entrepreneurship-related experience is acquired. One requirement to establish a successful enterprise is practical experience, which some scholars call “tacit knowledge” (Hellmann and Thiele, 2019). This cannot be learned through theoretical instruction because entrepreneurship is a highly practical activity. At present, EE in colleges pays attention to the combination of theoretical and practical education, so that students can experience the process of entrepreneurship and gain entrepreneurial knowledge through personal practice (Leitch and Harrison, 1999).

### Entrepreneurial Spirit

The entrepreneurial spirit (ES) concept in this study is taken from the 2017–2018 Global Entrepreneurship Monitor (GEM) report, which refers to the overall ES of a region, rather than the narrow sense (the entrepreneur’s subjective thoughts, personality, character, etc.). According to the 2017–2018 GEM Report, entrepreneurial awareness, entrepreneurial opportunity perception, and entrepreneurial self-efficacy are the three ES variables with the most significant effect. The first, entrepreneurial awareness is a powerful internal driving force for people to engage in entrepreneurial activities. The second, entrepreneurial opportunity perception refers to people’s subjective perception of the entrepreneurial opportunities around them. Finally, the third variable, entrepreneurial self-efficacy, refers to the degree of confidence that individuals have in their own ability to be successful entrepreneurs (Chen et al., 1998).

### Entrepreneurship Education and Policy in China

Countries worldwide attach great importance to entrepreneurship and development and have introduced policies to promote entrepreneurship. In Sweden, EPo implementation promotes entrepreneurship learning and culture more widely in higher education (Hoppe, 2016). In Turkey, structural support (whether supported by the structural system including private, public and NON-GOVERNMENTAL organizations) is vital for college students that are starting their own businesses (Turker and Senem Sonmez, 2008). Moreover, the Japanese government supports the establishment of an entrepreneurial culture by implementing policies that improve the climate for startup businesses (Ibata-Arens, 2008).

In 2014, Chinese Premier Li Keqiang put forward the slogan “Mass entrepreneurship and innovation” at the Summer Davos forum, and wrote it into the government work report the following year, which set off a wave of innovation and entrepreneurship in China. In 2018, the State Council of China issued the Opinions on Promoting High-quality Development of Innovation and Entrepreneurship and Building an Upgraded Version of Mass Entrepreneurship and Innovation, which emphasizes the promotion of mass entrepreneurship and innovation on a larger scale and at higher and deeper levels. EE in China started later than in western countries, but in recent years, it has also attracted much attention because of the country’s great efforts to promote innovation and entrepreneurship development. In contemporary China, formative instruction regarding innovation and entrepreneurship is relatively weak (Hong, 2018) owing to the lack of instructors with practical experience. Consequently, it is difficult for students to apply the principles of innovation and entrepreneurship successfully in practice. Some scholars have studied strategies to improve the efficacy of EE in colleges and universities using EPr as the principal vehicle (Wang, 2020). Some foreign scholars have put forward remedial measures and countermeasures for the challenges faced by Chinese students engaged in EE (Bell, 2020). Mok et al. (2019) show that China should consistently revise innovation policies, to adapt to the ever-changing environment and to support transformation into a more dynamic entrepreneurial nation. To stimulate college students’ willingness to engage in entrepreneurship and to alleviate the difficulty of entrepreneurship, the Central and local governments in China have successively issued policies supporting college students’ entrepreneurship, thus optimizing the policy environment for starting their own businesses. These include support policies, preferential tax policies, and other policies that encourage college graduates to start their own businesses.

### Hypotheses

Entrepreneurship policies have been shown to have a positive effect on entrepreneurship. For instance, research of Barreneche García’s (2013) shows that policies that encourage entrepreneurship stimulate the development of new enterprises. Autio and Rannikko (2016) believe that growth-oriented policy initiatives would affect the development of startup businesses. As previously stated, studies demonstrate that comprehensive EPos have a positive effect on EI. For example, the EI of individuals dwelling in rural areas can be improved through effective EPo implementation as well as education (Lin and Si, 2014). EPo and EE can further influence EI for e-business through indicators like entrepreneurial attitude, subjective norms, and perceived behavior control (Lai and To, 2020). According to this literature, EPo has a positive effect on entrepreneurship, whereas both EE and EPo have a positive effect on EI. These findings inform this study’s aim to explore the influence of EPo on EI. Therefore hypothesis 1 is posited.

**H1: EPos have a positive effect on EI.**

Since the implementation of EPos in Sweden, there has been an increase in the recognition that entrepreneurship is learned through practice (Hoppe, 2016). Opportunities for college students to practice entrepreneurship are limited. Most come from entrepreneurship competitions and courses, and they rely more on the guidance of schools and entrepreneurship teachers. Many countries have introduced new policies to promote entrepreneurial activities, with the focus on promoting entrepreneurship and the viability of start-up companies (Gilbert and Mcdougall, 2004). However, it is not clear whether EPos do promote EPr. Therefore, this study explores the influence of EPos on EPr and proposes hypothesis 2a.

**H2a: EPos have a positive effect on EPr.**

Governments can contribute by helping to raise awareness of successful entrepreneurial role models, removing bureaucratic barriers to start-ups, and reducing the social stigma of failure (Wang and Wong, 2004). Government support, EE, and the social climate all contribute to the identification of entrepreneurship opportunities among adults (Moon and Kim, 2018). The stronger the influence of entrepreneurship support on entrepreneurial self-efficacy, the more it will promote entrepreneurship (Malebana, 2017). Because there is little literature on the effect of EPos on ES, this study proposes hypothesis 2b.

**H2b: EPos have a positive effect on ES.**

Entrepreneurial practice is not only a learning process for students, but is also a demonstration of the very skills that they learn. Through this practical learning, students can better apply their knowledge and skills (Wee, 2004). Some scholars have highlighted the importance of students obtaining practical experience through entrepreneurial internship (Higgins et al., 2018). They emphasize the huge effect that practical training has on college students’ EI (Zhang et al., 2020), and that lack of practice is likely to affect students’ entrepreneurial success. Most of the literature finds that EE helps to improve EI. Nevertheless, some literature suggests that EE cannot improve EI (Chen et al., 2015). We think this is because the EE in their research only includes the study of theoretical knowledge without the combination with practice. Presently, in-depth studies concerning EPr are limited. Therefore, this study hopes to explore the influence of EPr on EI, and puts forward hypothesis 3a.

**H3a: EPr has a positive effect on EI.**

Entrepreneurial role models usually create entrepreneurial awareness, the first variable of ES. They stimulate entrepreneurial passion (Fellnhofer, 2017), thereby promoting EI. Notably, when the entrepreneurial atmosphere is strong, entrepreneurial role models can alleviate individuals’ fear of failure (Wyrwich et al., 2015). Previous studies on the influence of entrepreneurial role models on EI largely used the parental figure as the exemplar. Conversely, more recent studies have shown that entrepreneurial parents have no significant influence on their children’s entrepreneurial attitudes, and offspring are influenced by other factors (e.g., social pressure, personal views, etc.; Zapkau et al., 2015). In light of this, this study has selected the indicators of entrepreneurial awareness from the perspective of classmates or friends as entrepreneurial role models. The second variable, opportunity perception, forms a part of entrepreneurial readiness, which has a significant effect on EI (Schillo et al., 2016). The third variable, entrepreneurial self-efficacy is a prerequisite for the formation of EI (Zhang et al., 2020). Particular skills and experiences also affect entrepreneurship. Polin and Ehrman (2018) studied 500 veterans enrolled at Israeli universities and found that those with command experience were more willing to engage in entrepreneurship. Moreover, veterans from technological units usually showed greater interest in entrepreneurship than those from combat units. According to this literature, ES has a certain positive influence on EI. This study explores the influence of ES on EI and posits hypothesis 3b.

**H3b: ES has a positive effect on EI.**

The literature reviewed above, brings us to the conclusion that (1) in the EE process, EPo support will play its role through supporting students’ real EPr needs, giving them confidence in their entrepreneurship skills and finally improving their EI. (2) The large-scale implementation of EE will improve students’ entrepreneurial awareness, opportunity perception, and self-efficacy, in other words, it will improve the ES of the region, thereby strengthening the positive effect of EPo on EI.

**Figure 1:**
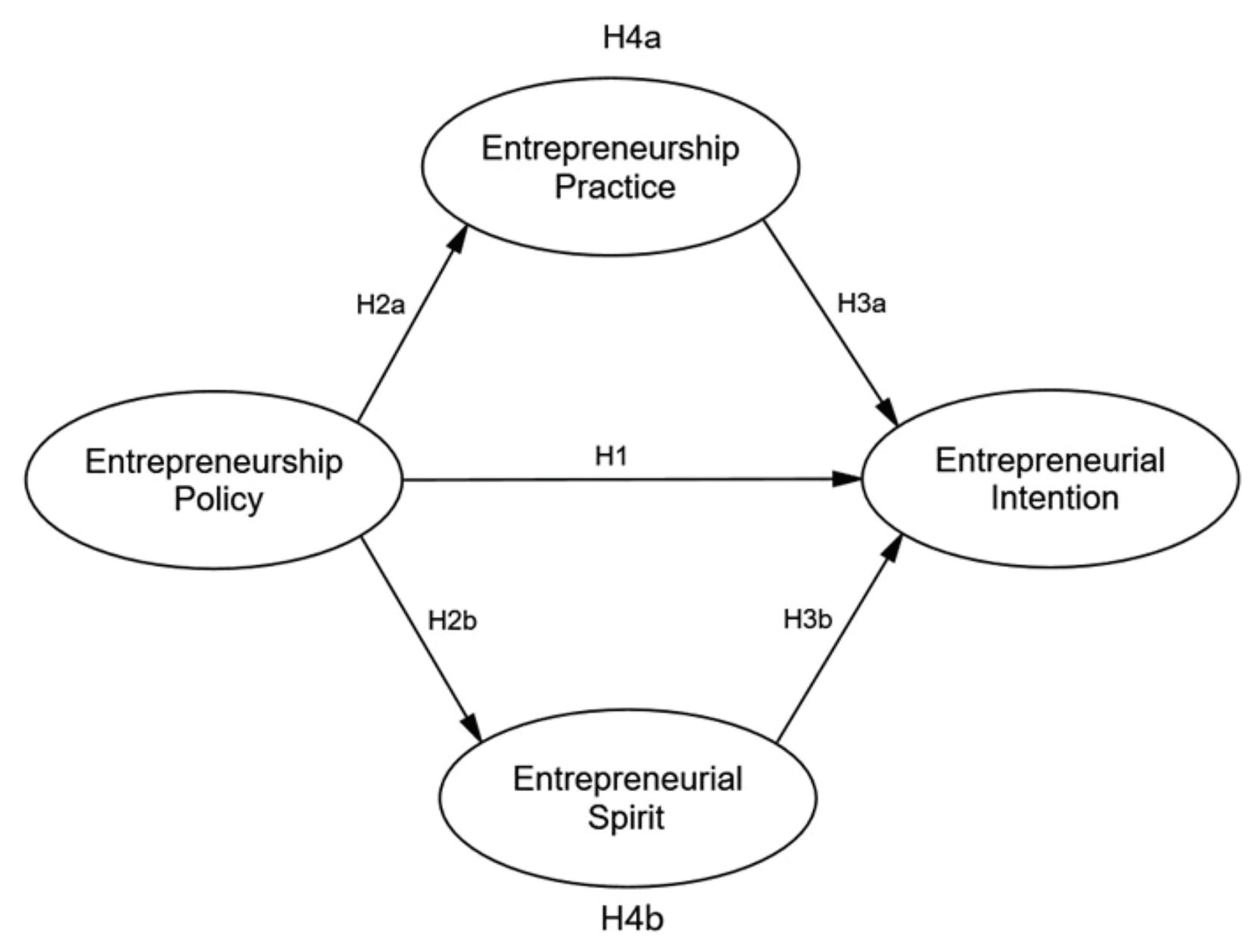
Research framework.

In summary, we think that EPr and ES are intermediaries and may mediate the stimulating effect of EPo and EI. The fourth hypothesis discusses these intermediary roles in the relationship between EPo and EI (Figure 1).

**H4a: EPr conciliates the positive effect of EPo on EI.**
**H4b: ES conciliates the positive effect of EPo on EI.**

## Materials And Methods

### Participants and Procedure

A separate survey was conducted in 2019 through online questionnaires. With 11.521 million permanent residents, Guangdong is one of the most open and economically dynamic regions in China. The dataset was based on the questionnaire survey and analysis of 384 students and graduates from 22 universities in the Guangdong Province. The decision was made to include undergraduate students and graduates who had attended university in the past 5 years to cover college students who are about to face employment problems and those who already are. The effective recovery rate of the questionnaire was about 91.2%. Table 1 shows the distribution of participants’ gender, major, EPr experience, graduate program, and entrepreneurial experience of family members. The respondents cover 11 majors, 62.2% are female and 37.8% male, 25.8% have practical experience in entrepreneurship, 13.3% are planning to start a business after graduation, and 25.8% have family members with experience in entrepreneurship.

**Table 1:** Descriptive statistics (*N* = 384).

Demographic variables	Dimension	Percentage (%)
Gender	Male	37.8
	Female	62.2
Major	Philosophy	0.8
	Economics	8.3
	Law	1.3
	Pedagogy	6.0
	Literature	3.9
	History	0.5
	Science	16.7
	Engineering	19.0
	Agronomy	0.5
	Medical	20.6
	Management	15.9
	Art	6.5
Entrepreneurial practice (EPr)	Yes	25.8
	No	74.2
Graduate program	Obtain employment	63.5
	Entrance for further study	21.9
	Entrepreneurship	13.3
	Else	1.3
Entrepreneurial experience of family members	Yes	25.8
	No	74.2

### Measures

Data were collected from students and graduates, through student class and alumni WeChat groups and emails. To ensure the accuracy of the scale, the questionnaire was reviewed and revised by experts in the research field and individuals or organizations with entrepreneurial experience. It comprised 31 questions, including multiple choice, five-point scale, and multiple-choice ranking. The content of the questionnaire included students’ basic information (gender, grade, etc.), their cognitive attitude toward entrepreneurship, the current state of innovation and EE at their respective schools, and an evaluation of their satisfaction. IBM SPSS software was used to test the reliability of the scale (see Table 2).

**Table 2:** The reliability and validation of the study model (*N* = 384).

	SFL	CR	AVE	α
Entrepreneurship Policy (Epo)		0.9603	0.858	0.96
The state has reduced or exempted taxes on college students’ self-employed businesses	0.930^***^			
Local governments have simplified the application process for university students to register their businesses	0.938^***^			
The School provides start-up funds for business (interest-free loans)	0.912^***^			
Society offers free training to guide entrepreneurship	0.925^***^			
Entrepreneurship Practice (Epr)		0.93	0.7688	0.929
Entrepreneurship practice has inside and outside the school guidance teachers	0.857^***^			
Entrepreneurial practice is supported by special venture funds	0.889^***^			
The school provides integrated entrepreneurial practice services	0.908^***^			
There is an independent entrepreneurship park for college students	0.852^***^			
Entrepreneurial Spirit (ES)		0.8466	0.6485	0.842
A classmate or friend has started a business in the past year	0.831^***^			
Business opportunities in your province are generally good	0.739^***^			
You think you have enough knowledge, skills and experience to start a business	0.842^***^			
Entrepreneurial Intention (EI)		/	/	/
I will try to set up a business in the following year	/			

SFL, standardized factor loading; CR, composite reliability; AVE, average variance extracted.

*** *p* < 0.001.

#### Entrepreneurship Policy Perception

Guided by the scope of this study, EPo was measured according to four indicators: “the state has reduced or exempted taxes on businesses founded by self-employed college students,” “local governments have simplified the application process for university students to register their businesses,” “the school provides start-up funds for business (interest-free loans),” and “society offers free training to guide entrepreneurship.” Responses to these prompts were scored with a maximum of five points. The questionnaire adopts a retrospective longitudinal design method and asks students about their perception of entrepreneurship policies in the past year. The Cronbach’s coefficient was 0.960.

#### Entrepreneurship Practice

Entrepreneurship practice was measured according to four indicators: “EPr has mentors both within the school, and without,” “EPr is supported by special venture funds,” “the school provides integrated EPr services,” and “there is an independent entrepreneurship park for college students.” Responses to these prompts were each scored with a maximum of five points. The Cronbach’s coefficient was 0.929.

#### Entrepreneurial Spirit

This study adopted the ES mentioned in the 2017–2018 GEM report, including the three most significant variables: entrepreneurial awareness, opportunity perception, and entrepreneurial self-efficacy. These three variables are reflected in the scale as follows. Entrepreneurial awareness: “Have any of your classmates or friends started a business in the past year?” Opportunity perception: “Are the entrepreneurial opportunities in your province generally good?” Entrepreneurial self-efficacy: “Do you think you have the knowledge, skills and experience to start a business?” Responses to these prompts were each scored with a maximum of five points. The Cronbach’s coefficient was 0.842.

#### Entrepreneurial Intention

Referring to previous literatures on measurement of EI (Kruegerjr et al., 2000; Díaz-García and Jiménez-Moreno, 2010; Zhang et al., 2014), entrepreneurial intention was measured according to one indicator: “I will set up a business in the following year”. Responses to this prompt were each scored with a maximum of five points.

## Results

### Measurement Model

This study used IBM SPSS Amos 24.0.0 and IBM SPSS 25.0 for the analysis. Furthermore, confirmatory factor analysis was used to evaluate the measurement model of the study variables (see Table 2). The composite reliability (CR) of the constructs exceeded 0.70 and confirmed the internal consistency reliability (Fornell and Larcker, 1981). The average variance extracted (AVE) value of the constructs exceeded the suggested benchmark of 0.50 and proved to have reasonable convergent validity (Segars, 1997). In addition, Table 3 shows that the square root of the AVE is higher than the value of the corresponding row and column, which provides support for discriminant validity (Fornell and Larcker, 1981; Hair et al., 2010). We examined the fit of a model in which indicators loaded on one factor, partly addressing common method variance (CMV) concerns regarding measures used in the study (Podsakoff and Organ, 1986). The chi-square difference between the two models was significant (*Δ*CMIN2 = 823.66, ΔDF = 3, *p* < 0.05), indicating that there was no significant CMV.

**Table 3:** Structural validity of the scale (*N* = 384).

	AVE	EPo	EPr	ES
EPo	0.858	**0.926**		
EPr	0.7688	0.782	**0.877**	
ES	0.6485	0.548	0.497	**0.805**
EI	/	0.469	0.334	0.059

Bold numbers are the square root of the AVE of each construct. Off diagonals are Pearson correlation of constructs.

As shown in Tables 4 and 5, *p* < 0.001, CMIN/DF = 3.054 < 5, RMSEA = 0.073 < 0.08, RMR = 0.043 < 0.05, GFI = 0.937 > 0.9, AGFI = 0.901 > 0.9, NFI = 0.965 > 0.9, CFI = 0.976 > 0.9, IFI = 0.976 > 0.9. Regarding the fitting index of the structural equation, all fitting indexes reach the standard, indicating that the scale has good structural validity (Hair et al., 2010).

**Table 4:** Structural validity of the scale.

Model	CMIN	DF	CMIN/DF	P
Default model	152.710	50	3.054	0.000

**Table 5:** Structural validity of the scale.

Model	RMSEA	RMR	GFI	AGFI	NFI	CFI	IFI
Default model	0.073	0.043	0.937	0.901	0.965	0.976	0.976

### Hypothesis Testing

IBM SPSS Amos 24.0.0 and IBM SPSS 25.0 were used to test the model and the bootstrap method (Bootstrap = 5,000) was used to test the relationship between variables (Kinnon, 2008; Taylor et al., 2008).

First, the data were analyzed through Amos. As shown in Table 6, (1) the direct effect of EPo on EI is 0.469 and *p* < 0.001. This indicates that the path has significant influence. Hypothesis 1 is supported. (2) The direct effect of EPo on EPr and ES is 0.784 and 0.553, respectively, and *p* < 0.001. This indicates that the path has significant influence. Hypotheses 2a and 2b are supported. (3) The direct effect of EPr on EI is 0.334 and *p* < 0.001, indicating that the path has significant influence. Hypothesis 3a is supported. (4) The direct effect of ES on EI is 0.059 and *p* > 0.001 indicating that the path has no significant influence. Hypothesis 3b is not supported.

**Table 6:** The direct effect of the research paths.

Path	Standardized estimate	S.E.	C.R.	P
H1: EPO-->EI	0.469	0.063	7.439	^***^
H2a: EPO-->EPR	0.784	0.042	17.503	^***^
H2b: EPO-->ES	0.553	0.057	10.467	^***^
H3a: EPR-->EI	0.334	0.062	5.72	^***^
H3b: ES-->EI	0.059	0.04	1.36	0.174

*** *p* < 0.001.

**Figure 2:**
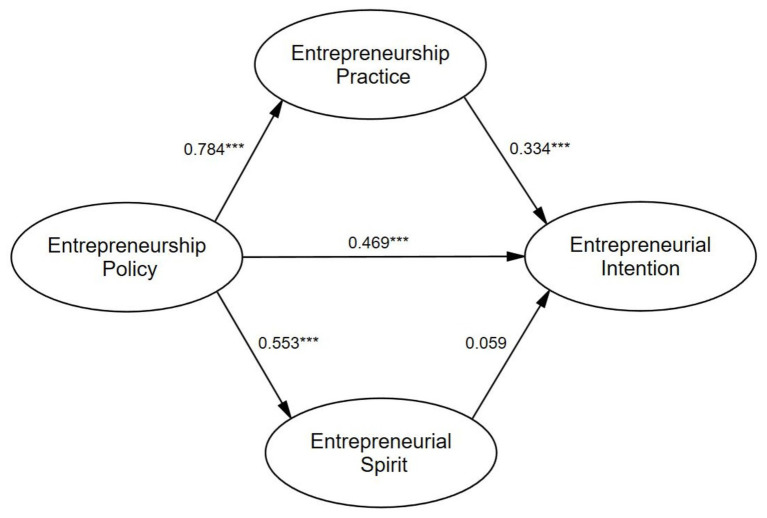
Standardized estimates of path coefficients of the SEM model (^***^p < 0.001).

The data were then analyzed by SPSS. The results presented in Tables 7 and 8 show that (1) the bootstrap 95% CI for the total indirect effects and the mediating effects of EPr does not contain zero. The total indirect effects account for 37.66% of the total effects and the mediating effects of EPr account for 32.61% of the total effects, indicating that EPr has a significant mediating effect. Hypothesis 4a is supported. (2) The bootstrap 95% CI for the mediating effects of ES does not contain zero. The mediating effects of ES account for 5.05% of the total effects. Hypothesis 4b is not supported. (3) The bootstrap 95% CI does not contain zero, indicating that the mediating effect of EPr is significantly greater than that of ES (Figure 2).

**Table 7:** Indirect effect of EPo on EI.

	Effect	Boot SE	BootLLCI	BootULCI	The proportion of the effect
TOTAL	0.274	0.053	0.177	0.386	37.66%
Epr	0.238	0.053	0.146	0.349	32.61%
ES	0.037	0.020	−0.001	0.078	5.05%
(C1)	0.201	0.060	0.094	0.327	/

Specific indirect effect contrast definitions: (C1) Epr minus ES.

**Table 8:** The direct effect, mediating effect and total effect of each variable on EI.

	Direct effects	Indirect effect	Total effect
EPo	0.454^***^	0.274^***^	0.729^***^
EPr	/	0.238^***^	/
ES	/	0.037	/

*** *p* < 0.001.

## Discussion

The results have shown that (1) EPo has a significant positive influence on EPr, ES, and EI. (2) EPr has a significant positive influence on EI. (3) ES does not have a significant positive influence on EI. (4) EPr has a remarkable intermediary role, whereas the ES intermediary effect was not significant. This indicates that EPo has a significant positive effect on EI through EPr.

### Theoretical Implications

This study has three theoretical implications.

First, it established a structural equation model of EPo, EPr, ES, and EI, and tested the intermediary roles of EPr and ES in the relationship between EPo and EI. The research verifies that EPr promotes EE; that EPo not only promotes the EE effect on college students directly, but also promotes it indirectly through EPr.

Second, some of this study’s conclusions are consistent with results of Higgins et al. (2018) and Zhang et al. (2020). In other words, EPr has a significant positive effect on college student’s EI after receiving EE, indicating that EPr is particularly important for generating EI. In addition, the test showed that EPo does not promote EI through ES, and ES has no significant promoting effect on EI. This is contrary to previous studies that improved the theory on the relationship between EI and its antecedent variables.

Finally, this study explored the role of the regional ES dimension. According to the GEM report, entrepreneurial awareness, opportunity perception, and self-efficacy are integrated into a new dimension of entrepreneurship, providing a new direction for research on the factors influencing EI in the future. Many scholars have studied the positive effect of entrepreneurship indicators on EI. For example, self-efficacy is considered an important prerequisite for EI, and entrepreneurial role models can only affect EI through self-efficacy (Krueger and Dickson, 1994). Zhao et al. (2005) confirm that entrepreneurial self-efficacy has a significant mediating effect on the preconditions for and EI of starting a business. Furthermore, empirical research of Liu et al. (2011) finds that identifying entrepreneurial opportunities is conducive to generating entrepreneurial will. We expected to find that ES influences EI significantly; however, this study’s results confirm that although ES does promote EI, the effect is not significant, and neither is its intermediary effect on the relationship between EPo and EI. This may be because the ES indicators are subjective and few entrepreneurial policies for these indicators have currently been implemented, which means that the current EE in colleges and universities cannot be adapted to such policies. It may also be that the scale maturity of the ES indicators used in this study is insufficient.

### Practical Implications

This study also has several practical implications for entrepreneurship policymakers, college students, and related institutions.

Previous research shows that students are dissatisfied with entrepreneurship learning, especially with how entrepreneurship theory learning and practice are combined with their majors (Huang et al., 2020). In the establishment of innovation and EE systems and the design of entrepreneurship courses, attention should be paid to the integration of textbook knowledge and practical experience, thus handling the contradiction between the discontinuity of tacit knowledge, entrepreneurial experience, and the continuity of classroom teaching requirements. Teachers with practical experience in entrepreneurship should be employed to guide students in EPr. The respective training institutions should also verify the qualifications of these instructors while strengthening their training (Ahmad et al., 2014). It may be worthwhile to explore avenues where teachers and students start businesses together if the conditions permit. Through EE, students can enhance their sense of social responsibility and voluntarily devote themselves to improving the entrepreneurship ecosystem within their region (Sánchez and Maldonado, 2019), to form a sustainable virtuous circle of “education – entrepreneurship – economic development.”

The government should design policies to encourage college students to start their own businesses and encourage the development of EE, establish a fair and green business environment, and introduce policies to strongly support students’ participation in entrepreneurship, such as providing financial support and interest-free loans. For students without a fixed income, start-up capital is an obstacle that is difficult to overcome. Rather than exposing them to this open market environment, direct financial policies would be more helpful (Lee, 2019; Wang and Huang, 2020). There is a need to create a cultural environment for innovation and entrepreneurship, cultivate students’ innovative and entrepreneurial thinking from an early age, eliminate their fear of entrepreneurship, and develop their entrepreneurial competency. Moreover, our research demonstrates that entrepreneurial policies are sufficient to promote (regional) ES (including entrepreneurial awareness, perceived opportunity, and entrepreneurial self-efficacy), which is recognized as a factor in promoting entrepreneurial willingness. However, these factors have no significant promoting effect on EI, which requires policymakers to consider whether redesigning policies in this area might produce better effects on EI.

## Conclusion

This study explores the intermediary effect of ES and EPr on the relationship between EPos and EI. It puts forward specific directions for improving entrepreneurship policies, while highlighting the importance of EPr. It also provides a fresh perspective for subsequent studies, which may further explore the role that ES plays.

This study regards entrepreneurship as a benign means to promote economic development. In fact, too much emphasis on economic utility and too little attention to social and responsibility may lead to the wrong direction in entrepreneurship education. Entrepreneurship is not suitable for everyone. While teaching entrepreneurship, we should understand the significance of education and learning (Verduijn and Berglund, 2019) and carry out critical entrepreneurship education (Komulainen et al., 2020). In the process of entrepreneurship education, there may be some conflict between entrepreneurial culture and traditional academic values, especially in the area of technology transfer (Gorovitz and Bok, 1983). However, in the process of the development of The Times, academic values, and entrepreneurial culture can gradually balance and run in (Ylijoki, 2003; Bullard, 2007), which is also an aspect that entrepreneurship education research should pay attention to. While pursuing material things, people should not go beyond academic boundaries. Policy makers should stop subsidizing the typical start-up and focus on the subset of businesses with growth potential (Shane, 2009), and so on. This is what entrepreneurship education research should pay attention to.

One limitation of this study is that the number of participants (*n* = 384) are small. However, the heterogeneity of the entrepreneurial ecosystem in different countries and cities (Kantis et al., 2020) will have different effects on each local entrepreneurial situation. Therefore, future research might explore these topics in other regions to make regional comparison more feasible and to propose practical measures to promote Students’ Entrepreneurial Intention differently in different regions. Another limitation is the retrospective longitudinal design method used in this study. The advantages of retrospective longitudinal design are: (1) simple design and less cost; (2) It can collect reliable information about the sequence of events, reconstruct the “career,” and propose some causal explanation. However, the disadvantages of this design are also obvious: (1) People may not be able to accurately recall their sensory experience, and distortion will occur intentionally or unintentionally in recall; (2) People tend to interpret experiences in terms of subsequent events, which makes it difficult to be objective. In addition, the EPo indicator setting standards are not well developed, and the measurement item of EI is relatively single, so we will optimize them in future studies.

## Data Availability Statement

The datasets analyzed in this article are not publicly available. Requests to access the datasets should be directed to 673336392@qq.com.

## Ethics Statement

Ethics approval for this research was not required as per institutional and national guidelines. Consent from all research participants was obtained by virtue of survey completion.

## Author Contributions

YH, SW, and LA described and developed the review and the hypothesis. YH, JW, and PW was involved in the data collection process. SW, YC, and LA performed the analysis, interpretation of the results and formulated the main conclusions. LA, JW, and PW formulated the study limitations and future directions for research. All the authors’ help editing and formatting the paper. All authors contributed to the article and approved the submitted version.

### Conflict of Interest

The authors declare that the research was conducted in the absence of any commercial or financial relationships that could be construed as a potential conflict of interest.
